# Augmentation-related brain plasticity

**DOI:** 10.3389/fnsys.2014.00109

**Published:** 2014-06-11

**Authors:** Giovanni Di Pino, Angelo Maravita, Loredana Zollo, Eugenio Guglielmelli, Vincenzo Di Lazzaro

**Affiliations:** ^1^Institute of Neurology and Fondazione Alberto Sordi - Research Institute for Ageing, Campus Bio Medico University of RomeRome, Italy; ^2^Laboratory of Biomedical Robotics and Biomicrosystems CIR - Centre for Integrated Research, Campus Bio Medico University of RomeRome, Italy; ^3^Department of Psycology, Università di Milano-BicoccaMilano, Italy

**Keywords:** supernumerary limbs, sensory substitution, cognitive enhancement, embodiment, brain machine interface (BMI), cross-modal plasticity, hand prostheses, sensorimotor abilities

## Abstract

Today, the anthropomorphism of the tools and the development of neural interfaces require reconsidering the concept of human-tools interaction in the framework of human augmentation. This review analyses the plastic process that the brain undergoes when it comes into contact with augmenting artificial sensors and effectors and, on the other hand, the changes that the use of external augmenting devices produces in the brain. Hitherto, few studies investigated the neural correlates of augmentation, but clues on it can be borrowed from logically-related paradigms: sensorimotor training, cognitive enhancement, cross-modal plasticity, sensorimotor functional substitution, use and embodiment of tools. Augmentation modifies function and structure of a number of areas, i.e., primary sensory cortices shape their receptive fields to become sensitive to novel inputs. Motor areas adapt the neuroprosthesis representation firing-rate to refine kinematics. As for normal motor outputs, the learning process recruits motor and premotor cortices and the acquisition of proficiency decreases attentional recruitment, focuses the activity on sensorimotor areas and increases the basal ganglia drive on the cortex. Augmentation deeply relies on the frontoparietal network. In particular, premotor cortex is involved in learning the control of an external effector and owns the tool motor representation, while the intraparietal sulcus extracts its visual features. In these areas, multisensory integration neurons enlarge their receptive fields to embody supernumerary limbs. For operating an anthropomorphic neuroprosthesis, the mirror system is required to understand the meaning of the action, the cerebellum for the formation of its internal model and the insula for its interoception. In conclusion, anthropomorphic sensorized devices can provide the critical sensory afferences to evolve the exploitation of tools through their embodiment, reshaping the body representation and the sense of the self.

## Introduction

Humans have always tried to augment their able-body abilities with the help of tools; lenses have been exploited to see further afield or to look at tiny objects, vehicles to travel long distances quicker and pincers and tongs to tightly manipulate objects. Tools use is a unique feature that humans and primates share, whit a loud influence in evolutionary processes, allowing them to push their abilities beyond the boundaries set by evolution and better interact with the environment (Ambrose, 2001).

Today’s achievements in biological sciences and engineering might need to reconsider the concept of human-tools interaction in the framework of human augmentation. Human augmentation is a newborn domain of investigation that aims to exploit methodologies proper of medical therapeutic intervention and rehabilitative medicine, such as strategies, drugs and external artificial devices originally designed to compensate for the loss of functions, to increase physical and cognitive ability of able-bodied individuals, beyond the level characteristic of the normal physiological health status. Hence, the health status of the population targeted by the intervention critically sets the difference between rehabilitative functional restoration and human augmentation.

Actually, the idea of augmenting human abilities is not brand new, since, for instance, almost half a century ago, Von Gierke stated that it was among the goals of bionics “to extend man’s physical and intellectual capabilities by prosthetic devices in the most general sense” (Von Gierke, 1970). However, more recently, the field of human augmentation started to capture the attention of scientists worldwide and to raise the level of awareness on its ethical and societal implications (for a deep analysis of ethical and societal implications of human augmentation see Clark, 2007; Blanke and Aspell, 2009). Indeed, several features of modern functional prostheses signed a sharp discontinuity with any device previously aimed at enhancing the ability of able-bodied: (i) the achieved high level of anthropomorphism of the tool; (ii) the intimate contact that the tool establishes with the user; and (iii) the absence of bottlenecks in the flow of information from the brain to the environment on which the tool operates. In summary, in recent years we are assisting to the first true attempts to create Hybrid Bionic Systems (HBSs), containing both technical and biological components arranged in a tight complex, where the robotic artifact is directly interfaced with the brain of a human being (Dario et al., 1993). This scenario encompasses, for example, modern cybernetic hand prostheses that exploit neural signals taken by electrodes implanted invasively into the cortex or into peripheral nerves to decode the user’s intention of movement (Hochberg et al., 2006; Rossini et al., 2010). This modern approach could eventually lead to an unprecedented degree of man-machine integration.

The development of invasive (Lebedev and Nicolelis, 2006) and non-invasive (Birbaumer and Cohen, 2007) Brain-Machine Interfaces (BMI) and neural interfaces with peripheral nerves (Navarro et al., 2005) allowed to bypass the activity of the muscles and the physical transduction of cutaneous/proprioceptive sensory organs, while directly picking up motor signals from, or delivery sensory inputs to the nervous system. Hitherto, this has been the main determinant of the functional continuity between man and machines and represents the biggest milestone in the enquiry of the human augmentation. Prostheses that the user operates through neural interfaces are often named neuroprostheses. Although this term literally means a prosthesis for replacing a function of the nervous system, as it was originally conceived in the domain of cochlear implant (Terr et al., 1987) and in the stimulation of the sacral roots for bladder control (Brindley, 1974), it was lately used for motor functional electrical stimulation (Popovic et al., 2002) and more recently started to be used also for prostheses interfaced with the nervous system (Lebedev and Nicolelis, 2006). This extended meaning seems to be sufficiently accurate in operational terms and will be use from now on.

The recent advancements in robotics and BMIs are key enabling technologies for the creation of an augmented bionic man. However, can humans adapt themselves to effectively control external additional limbs and other body parts by learning and integrating them into a new sensorimotor representation? In the attempt to answer the question, it might be helpful to make a simple parallelism with the operation of adding a printer or an external hard-drive to our personal computer. The compatibility of the hardware is *conditio sine qua non, moreover* printer and laptop must share, for instance, the same I/O port. However, for a new device to be proficiently controlled the presence of its “representation” in the software that manages external devices is mandatory. In the case the software was not originally designed to allocate that device, it needs to be reprogrammed, often by means of supplemental drivers that plastically adapt it to the new condition. Analogously, human brain can be trained to expand its motor control to a supernumerary, artificial limb or else to receive sensory information from an external accessory sensor and plastically embed them into its representation of the body.

Most of the advancements in the field of human-machine interface have been achieved by pursuing two strategies in parallel: on one hand, the development of devices for restoring sensorimotor functions in disabled and, on the other hand, the development of devices for augmenting sensorimotor capabilities of able-bodied, allowing for instance to operate in impervious environments. However, it is worth considering that a further possible field of application of such technology, located in between rehabilitative functional restoration and human augmentation, is aging. The augmenting technology can be aimed indeed at supporting sensorimotor and cognitive abilities that are lost day-by-day with normal aging. The existence of a net border between therapeutic and augmenting applications seems to be overestimated, since the continuous distribution of individual human performance and its extreme variability could make exploitable for augmentation what has been developed for restoration and vice versa. Along this line, as we will see in the following paragraphs, most brain processes subtending functional restoration match the ones subtending augmentation and can be exploited to understand this phenomenon.

This review is aimed to analyze the neural correlates of human augmentation and, in particular, the plastic process that the brain undergoes when it comes into contact with artificial sensors and effectors meant as external aids and, on the other hand, the changes that the use of external augmenting devices produces in the brain.

## Brain plasticity: general considerations

Brain plasticity is the ability of the brain to adapt its structural and functional connectivity in response to an external condition promoting a novel function, or a new way to perform an old function, or else the suppression of a sensorimotor ability. Plasticity has widely been considered the neural substrate of early development (Hensch, 2005), of the acquisition of new skills (Pascual-Leone et al., 1995) and of the recovery from brain injuries (Chen et al., 2002). It may be intended as an inner property of neural networks that results from the exposure of the system to physiological or pathological conditions (Pascual-Leone et al., 2005).

The Hebb and Paillard’s theoretical hypothesis of brain plasticity (Hebb, 1949; Paillard, 1976), postulating that a long lasting strengthening of the connection between two neurons is induced by the simultaneous activation of those cells, has found its neural correlate in the phenomenon of associative learning mediated by long-term potentiation/depression (Bliss and Lomo, 1973; Bailey and Kandel, 2008).

Most of the findings regarding brain plasticity first came from lesion study in animals and, later on, from non-invasive functional imaging in humans. Plastic changes have been widely described in sensorimotor cortices that undergo deep remodeling with an extension of cortical representation of the still functionally active projections (Kaas, 1991). An initial unmasking of already present, but inactive, connections is the effect of increased cortical excitability due to a reduction of GABA-mediated inhibition (Jones, 1993) and an increase of NMDA-mediated activation (Buonomano and Merzenich, 1998). More stable changes often underlie structural modifications, characterized by axonal regeneration and sprouting (Kaas, 1991).

Hitherto, the amount of studies on ability augmentation, nearly all in animals, which investigating the recruited neural networks and/or on the plastic processes involved, are not enough to build a comprehensive body of knowledge on this topic. However, clues about augmentation-related plasticity can be obtained by borrowing insights gained from similar logically-related paradigms, i.e., sensorimotor training, cognitive enhancement, cross-modal plasticity, sensory and motor output functional substitution, use of tools and embodiment.

For instance, sensorimotor abilities can be enhanced by specific training and are boosted in athletes, while cognitive training enhances memory and attentive functions in healthy and brain-damaged, or aged, individuals. Artificial devices for etero-modal sensory substitution are exploited in the deaf and blind and rely on cross-modal plasticity, but the same devices can be exploited for sensory augmentation in sighted and normal hearing people. Motor augmentation, as well as motor functional substitution, involves the use of external effectors, including prostheses. Their optimal functionality relays on their integration in the user body schema, like any other kind of hand-held tool. Starting from those considerations, we will briefly describe the aforementioned paradigms and revise their implications for augmentation-related plasticity.

## Sensorimotor training-induced plasticity

Several effects of sensory motor training have been demonstrated in both the sensory and the motor domains, as well as at cellular level and at the level of whole brain areas or brain networks. Within the sensory domain, the acquisition or improvement of sensory functions is accompanied by plastic changes in the brain. Sensory discrimination training is able to induce changes in primary sensory cortices. Frequency discrimination training in adult owl monkeys results in increased performance and an enlargement of the stimulated skin representation in the primary somatosensory cortex, where the receptive fields of sensory neurons were significantly expanded (Recanzone et al., 1992). For example, monkeys trained in a visual orientation task showed a refined tuning of V1 neurons towards the trained orientation (Schoups et al., 2001).

Within the motor domain, a huge body of literature is devoted to the motor system plasticity induced by training. In rats, practicing a reaching task produced an enlarged representation of the wrist and digit movements in the primary motor cortex (M1) with an increase of the number of synapses per neurons (Kleim et al., 2002). The improvement seen in a reaction task can be well inferred from the activity of the motor neuronal ensemble in charge of the task (Laubach et al., 2000). In awake monkeys, skills acquisition modulates the activity of M1 neurons as assessed through cortical invasive recordings (Germain and Lamarre, 1993). Furthermore, the enlargement of M1 depends more on motor skill acquisition (Nudo et al., 1996) than on the simple repetitive use (Plautz et al., 2000). Long-term training-induced plastic changes in neuronal properties seem to be the substrate for the internal storage of motor skills (Matsuzaka et al., 2007). M1 changes during motor sequence learning, as evidenced by functional magnetic resonance imaging (fMRI), present an initial reduced area of M1 activation, following short-term repetition, but a progressive increased of the extension of M1 activation following motor training. Such a pattern of activation may be the neural substrate underpinning a three-phase motor skill acquisition: initial habituation, consolidation and long-lasting plasticity (Karni et al., 1998). A model that has been largely used to assess sensorimotor plasticity is that of studying the brain of people that hold (or else acquire) peculiar sensorimotor skills, such as sport or music expertise. For example, learning a one-hand piano exercise produces an enlargement of the motor representation of the hand and a facilitation of the corticospinal tract devoted to the muscles of the trained fingers (Pascual-Leone et al., 1995). Expert tennis players have an asymmetry of hand motor cortex with an enlarged representations and increased motor cortex excitability as evaluated by measuring the threshold for motor-evoked potential (MEP) after transcranial brain stimulation, in the cortex controlateral to the hand using the racket (Pearce et al., 2000). Plastic changes take place in the somatosensory system as well. In violin players, the somatosensory cortical representation of the fingers used to play the strings are enlarged and the amount of enlargement correlates with the years of practice (Elbert et al., 1995).

As far as the neurobiological mechanisms of sensorimotor plasticity, evidence from both animal (Rioult-Pedotti et al., 1998, 2000) and human (Ziemann et al., 2004) studies attributes training-induced motor plasticity to long-term potentiation (LTP)-like mechanisms involving the synaptic strength of cortical horizontal connections. However, structural plasticity, in parallel with the modulation of synaptic strength, plays a crucial role even after a few days of training. In humans, learning to juggle induced a bilateral increase in the gray matter of the occipito-temporal cortex, especially in the middle temporal motion-sensitive area (Draganski et al., 2004), after only a week of practice. Such plastic changes were no more present after the training ceased, although the performance did not decrease (Driemeyer et al., 2008). Structural modifications have been reported also for the white matter underlying the intraparietal sulcus (Scholz et al., 2009). Moreover, exercise showed to stimulate neurogenesis in the dentate gyrus of the hippocampus in mice and humans (Pereira et al., 2007).

Sensorimotor plasticity also manifests as a change in the pattern of activation of different brain areas and circuits. Motor skill acquisition recruits brain regions that are not recruited during simple motor task execution (Grafton et al., 1992). Several factors influence which network is recruited by practice, such as the specific task domain and the behavioral and cognitive load required. In general, practice of sensorimotor tasks determines an increased reliance on sensorimotor areas and a decreased recruitment of attentional control exerted by prefrontal, anterior cingulate and posterior parietal cortex (Kelly and Garavan, 2005). Motor practice not only affects the pattern of brain activation involved in the execution of the movement, but also its preparation. It has been shown that during stroke preparation expert golf players, compared to novices, show higher levels of activity in areas involved in visuomotor integration (superior parietal lobule, the dorsal lateral premotor cortex and the occipital area), and decreased activation in attentional/emotional basal ganglia and limbic structures (Milton et al., 2007). In the same paradigm, electroencephalographic (EEG) recordings demonstrated higher frontal theta and parietal alpha power, probably due to attention focusing for sensory processing (Baumeister et al., 2008). Skilled motor performance refines also the activity of the mirror system and goes in parallel with enhanced ability to anticipate the outcome of actions executed by others by resonant motor activation (Aglioti et al., 2008).

In summary, augmentation of sensorimotor skills, and sensorimotor training produce, an enhancement of performance, which is paralleled by specific neurobiological changes in the brain tissue and a change in the pattern of cortical activity, mainly by the focalization of brain activity on sensorimotor cortex both during movement execution and preparation (Yarrow et al., 2009).

## Cognitive enhancement

Cognitive enhancement is the attempt to improve cognitive functions (memory, working memory, attention, fluid intelligence) through training, psychological strategies, drugs or other medical interventions and last, but not least, external technological supports. Today’s human augmentation targets cognitive enhancement *per se*, or can affect it as a consequence of sensorimotor augmentation. However, pursuing cognitive enhancement can be considered a foundational goal of humans. For instance, the aim of education always went beyond the mere learning of specific information; even pencil and paper can be regarded as primitive forms of external memory enhancement, while the use of nicotine and caffeine to focus attention, increase alertness and reduce the sense of fatigue can be dated far back in time (Bostrom and Sandberg, 2009).

The amount and quality of the stimuli offered by the environment are main determinants of cognitive development (Taffoni et al., 2014) and can be used to burst cognition. Indeed, in rats, an enriched environment produces an improvement of spatial memory and increases neurogenesis in the dentate gyrus (Nilsson et al., 1999) in a comparable way to chronic cholinergic treatment (Murphy et al., 2006). Also sensorimotor exercise, further than in sensorimotor processes, has a deep impact on cognition and promotes brain plasticity by modulating regional bloody flow and neurotrophic support, especially by releasing brain derived neurotrophic factor (BDNF; Vaynman and Gomez-Pinilla, 2005).

Cognitive enhancement by can be achieved with drugs. These molecules mostly target neurotransmitters of ascending systems from the brainstem nuclei, and have been directed to treat cognitive impairments of attention deficit hyperactivity disorder (ADHD), Parkinson’s disease, schizophrenia, while acetylcholinesterase inhibitors are currently used as a the therapy in Alzheimer’s disease (Husain and Mehta, 2011). Memory enhancing drugs are of two main classes: (i) LTP inducing drugs, mostly modulating AMPA (α-amino-3-hydroxy-5-methyl-4-isoxazole propionic acid receptors); and (ii) molecules increasing the cAMP response element-binding protein that enhances synapses, stabilizing proteins to allow memory consolidation (Farah et al., 2004). Effects of those drugs can go beyond the cognitive domains and influence non-cognitive symptoms of those clinical conditions. In this line, the acetylcholinesterase inhibitor rivastigmine reversed the abnormality of sensorimotor integration, as evaluated by testing short-latency afferent inhibition, in patients affected by Alzheimer’s disease (Di Lazzaro et al., 2005).

It is worth noting that memory enhancement techniques have been developed to counteract memory decline of Alzheimer’s and other neurodegenerative disorders, but they are currently extended to the healthy elder population in order to counteract age-related involution. Here again the borders between therapy and augmentation are weak. Similarly, methylphenidate (Ritalin), a catecolamine-like drug that represents the treatment of choice for ADHD and is known to improve cognitive performance also in healthy volunteers, is largely assumed even by children without diagnosed AHDH (Farah et al., 2004). The use of drugs for cognitive enhancement produces structural and functional changes in the brain. In healthy volunteers, the cognitive improvement seen after a single dose of modafinil, a monoamminergic stimulator, goes in parallel with an increased functional connectivity at rest in the anterior cingulate cortex, part of the left fronto-parietal control network and in the bilateral occipito-parietal node of the dorsal attention network (Esposito et al., 2013). Structural changes have been also described following drug treatment. In a rat model of stroke the administration of D-amphetamine induced an amelioration of motor and working memory performance and a significant increase of neurites growth and synaptogenesis in the neocortex (Stroemer et al., 1998).

Does superior memory ability rely on higher Q.I. or particularly developed brain structures or alternatively does it mostly rely on a specific functional engaging strategy? Evidence is in favor of the latter. During memory tasks, people with exceptional memory activate different neural networks involved in spatial learning and navigation, especially the hippocampus. This luckily reflects a “method of loci” memorizing strategy based on the association of each object to memorize with a location in an imagined physical pathway (Maguire et al., 2002). In healthy subjects, spatial memory can be dramatically increased by training, as happens for London taxi drivers that aim to acquire the license. A voxel-based morphometry study documented an increased gray-matter volume of the posterior hippocampus, which correlates with years of works and that can represent the plastic substrate for the allocation of spatial representations (Maguire et al., 2000).

Several attempts to achieve cognitive enhancement target the working memory. Working memory is the ability of retaining information over a brief time. It plays a pivotal role in most cognitive functions and is strictly linked with inhibitory functions, reasoning and intentional allocation of self-attention (Klingberg, 2010). In particular, spatial working memory improvement exerted by methylphenidate has been associated to a task-related activity refinement in the posterior parietal cortex and dorsolateral prefrontal cortex, especially on the left hemisphere, (Mehta et al., 2000); the former locus seems to relate to online organization and storage of information and the latter to their active manipulation and monitoring. Working memory can be enhanced by training. Several training programs have been developed, for instance computerized training devoted to ADHD children (Klingberg et al., 2005), that showed long-term efficacy (Holmes et al., 2009). Training working memory shapes the brain (Klingberg, 2010) by increasing the activity in the middle frontal gyrus, in the superior and inferior parietal cortices (Olesen et al., 2004) and in the caudate nucleus (Dahlin et al., 2008) and decreasing the number of cortical D1 dopamine receptors (McNab et al., 2009). Training affecting the intraparietal-prefrontal network yields effects that are not modality specific and that can be transferred to any different task requiring working memory (Thorell et al., 2009). Indeed, the positive effect in the retention of instrumental activities of daily-living in older adults produced by cognitive training have been documented even after 5 years from the initial intervention (Willis et al., 2006). The extension of performance improvement to untrained domains has a deep impact in the translation to human cognitive augmentation.

Being brain plasticity the base of cognitive enhancement, training programs specifically designed to target its mechanisms gave very promising results. It has been shown in healthy adults over 60, in whom a training program comprising stimulus recognition, discrimination, sequencing, and memory tasks under strict attentional control, high reward, and novelty has been used to target age-related degraded sensory processing and the down-regulation of neuromodulatory control nuclei. The training produced a memory improvement, which generalized to untrained tasks and was maintained over 3 months (Mahncke et al., 2006). Similar findings were demonstrated in children affected by dyslexia. A program composed by auditory and oral language training produced an increased activity during language processing in the right fronto-temporal regions and anterior cingulate cortex and, similarly to unaffected children, in the left inferior frontal gyrus and left temporo-parietal cortex. Activity in the latter area positively correlated with language recovery (Temple et al., 2003).

Currently, the wide diffusion of computer and videogames-based technology for cognitive training gives the opportunity to proficiently self-train cognitive abilities (Jak et al., 2013). Recently, a multitasking performance training videogame has proved effective in restoring, in elder adults, the same brain activity pattern found in younger controls, with an increase in the midline EEG theta band power over the frontal regions and a higher coherence with the posterior regions. EEG changes predicted the improvement of sustained attention and working memory and their maintenance after 6 months (Anguera et al., 2013).

Meditation, in its various forms, is a kind of mental training with a diverse and long-lasting history, that can be exploited as a strategy for cognitive enhancement (So and Orme-Johnson, 2001). It is able to enhance pre-attentive processes, as evidenced by an increase of the amplitude of auditory mismatch negativity waves (Srinivasan and Baijal, 2007) and of the functional activity of anterior cingulate cortex, prefrontal cortex, hippocampus and insula (Lazar et al., 2000; Farb et al., 2007; Hölzel et al., 2008; Lutz et al., 2008). Indeed, meditation induces short and long-term plasticity. High amplitude gamma-band activity has been described during meditation, especially over the lateral fronto-parietal electrodes, and long-distance phase-synchrony, while resting state EEG shows higher gamma/theta+alpha ratio which burst during meditation and persists after it (Lutz et al., 2004). Several studies demonstrated that meditation is able to induce structural changes especially in the prefrontal cortex, hippocampus and the right anterior insula (Lazar et al., 2005; Pagnoni and Cekic, 2007; Hölzel et al., 2008; Luders et al., 2009). Those areas are involved in the regulation of emotions and in their integration with cognition. Changes have been also evidenced in the brainstem (Vestergaard-Poulsen et al., 2009). Recently, a longitudinal follow-up after 8 weeks of meditation documented an increase of gray matter in the left hippocampus, in the posterior cingulate cortex, the temporo-parietal junction, and in the cerebellum (Hölzel et al., 2011).

Brain activity can be voluntarily modulated to pursue cognitive enhancement with the help of neurofeedback, an operant conditioning paradigm, in which participants exploit a feedback of their brain electrical activity to learn to influence it. Several neurofeedback protocols have been attempted so far. For instance, increase of beta/theta+alpha ratio (Rasey et al., 1995) and increase of sensorimotor (12–15 Hz) rhythm to achieve improvement of working memory and attention (Vernon et al., 2003) or perceptual sensitivity and reduced omission errors (Egner and Gruzelier, 2004), beta rhythm to improve reaction time (Egner and Gruzelier, 2004), increase peak of alpha to improve speed of processing and executive function (Angelakis et al., 2007) and frontal-midline theta activity to improve attention and working memory (Wang and Hsieh, 2013). However, changes of EEG rhythm and improvement of cognitive performance have to be taken with caution: in subject exposed to sham neurofeedback the sole attempt to control a bar, that they believed to be driven by EEG rhythm, produced a wide engagement of fronto-parietal and cingulo-opercular network, which are known to be involved in cognitive control (Ninaus et al., 2013).

Further than with the above mentioned drugs, trainings and strategies, cognitive enhancement can be achieved through external technological support and invasive brain stimulation. For instance, computer based memory aids are interactive diaries that can be embedded in portable or wearable devices and that help patients to remind everyday tasks such as calling a relative or taking a medication (Schulze, 2004). As regard as the stimulation, deep brain stimulation of the septal nucleus (Jiang et al., 1997) and high frequency stimulation of caudate and striatum (Williams and Eskandar, 2006) ameliorate learning and memory in humans and rodents, similarly to vagal nerve stimulation (Clark et al., 1999). Coupled cortical stimulating/recordings arrays can be exploited to trigger and support cortical plasticity (Jackson et al., 2006) and to burst inter-regional functional connectivity at the base of cognitive enhancement, or to substitute lost white matter in demyelinating lesions and subcortical atrophies (Serruya and Kahana, 2008). In theory, cognitive enhancement could, one day, completely rely on external modules. Indeed, artificially interfacing a cortical area with a different one is not very dissimilar from interfacing it with external ectopic (namely located in an abnormal position or environment respecting to the one for which they were originally developed) neural modules. Technology could provide surrogates of cortical or basal ganglia circuitries externally grown *in vitro* (Pfister et al., 2007) or hybrid neuron-chips where neurons grow in a silico support (Zeck and Fromherz, 2001; Serruya and Kahana, 2008).

In conclusion, cognitive enhancement can be achieved through appropriate training strategies and drugs and it mostly relies on plastic processes modulating neurotrasmitters ascending systems and involving the frontoparietal network and, in the case of working memory enhancement, the hippocampus.

## Cross-modal plasticity and sensory substitution

Literature on plasticity across the systems has been focused on the investigation of the changes that a disrupted sensory modality evokes on different sensorial networks, as occurring in sensory-deprived animals and humans. However, cross-modal plasticity can be considered as an example of the propensity of some brain areas to manage functions that they have not been originally aimed at.

Reading Braille produces an expansion of the S1 representation of the reading fingers in the blind (Pascual-Leone and Torres, 1993), but in parallel, a task-specific activation of V1 (Sadato et al., 1996), which is critically not present if the hand is used for motor tasks others than Braille reading (Gizewski et al., 2003).

Although basic parameters of spared sensory functions, such as visual contrast sensitivity (Finney and Dobkins, 2001), absolute auditory or tactile threshold (Niemeyer and Starlinger, 1981) may not be affected, cross-modal plasticity results in more complex behavioral advantages, as in the case of blind that process sounds faster and better and have enhanced tactile accuracy (Roder and Neville, 2003). This sustains the localization of this plasticity to be primarily in associative cortices. In this line, functional neuroimaging studies documented an increased recruitment of posterior superior temporal sulcus and inferior parietal lobe in the processing of stimuli processed by spared senses in the blind (Büchel et al., 1998) and deaf (Bavelier et al., 2001). However, even primary sensory areas are targets of cross-modal plasticity and in animals the artificial transposition of fibers from the retina to S1 makes S1 responding to light stimulation (Métin and Frost, 1989), while disruptive transcranial magnetic stimulation (TMS) targeting V1 cortex impairs a tactile discrimination task in blind humans (Cohen et al., 1997).

To understand the impact of cross-modal plasticity in augmentation, a first question is whether cross-modal plasticity is active only in response to brain damage or sensory deprivation. Intracortical invasive recordings in animals documented activity in V1 evoked by non-visual stimuli also in non-deprived animals (Murata et al., 1965) and tactile stimulation enhance V1 activity of healthy subjects (Macaluso et al., 2000), thus raising the intriguing possibility that even primary sensory cortices, in physiological conditions, are not completely unimodal. Indeed data support the idea of the existence of heteromodal connection between primary sensory cortices, as found between primary visual and somatosensory cortices in the monkey. Furthermore, connections between primary sensory areas through multisensory cortices may provide feedback projections that may enhance the response to a stimulus presented in one sensory modality when a spatially-temporally congruent stimulus is delivered in a different sensory modality (Macaluso and Maravita, 2010).

The mechanisms at play during cross-modal plasticity are likely the same of intra-modal plasticity and involve changes in local connectivity that warrant for the rearrangements of sensory maps, stabilization of transient long-range connections during development and changes in cortico-cortical feedback (Bavelier and Neville, 2002) and are mostly driven by activity-dependent inputs competition. Those changes are easier during childhood, but still possible along the adult life and may be the consequences of plasticity of subcortical structures, as in the thalamus and/or brainstem nuclei (Jones and Pons, 1998). Cortical feedback exerts also an important role in determining cross-modal changes, involving direct long range connections between primary sensory areas or connections through associative cortices, in line with the finding of enhanced fMRI connectivity between visual and parietal areas in deaf individuals (Bavelier et al., 2000).

Taking together the premises that sensory brain areas that are classically considered unimodal may be not strictly unimodal and that the mechanisms behind cross-modal plasticity are mostly the same at the base of intra-modal plasticity, would raise the hypothesis that the main determinants of plasticity are the features of the experienced stimulus, its timing along the development of the nervous system and the neurobiological features of the targeted system (Bavelier and Neville, 2002). This would happen mostly independently from any a priori restriction related to the modality of the stimulus, allowing therefore to easily conceive, and practically achieve, an artificial heteromodal sensory substitution.

It deserves to be mentioned that cross-modal plasticity could, in some circumstances, be detrimental for sensory-replacement implants, because it sustains a rewiring of the target orphan cortex from areas controlling other modalities, that could compete with inputs coming from the implant (Lee et al., 2001).

Little evidence sustains so far that also the motor system can undergo changes prompted by the incorporation of functions of different modalities: for instance motor cortex representation of the reading fingers is enhanced in Braille-readers far more than the extent only ascribable to its mere increased use (Pascual-Leone et al., 1993). As far as the effect of multi-modal plasticity induced by the introduction of devices offering new motor efferences, it would be extremely interesting to look at widespread brain plastic modifications in primates experiencing the control of a third arm.

Prostheses designed for sensory substitution, rely on crossmodal plasticity where afferences from a sensory modality are employed to guide the accomplishment of tasks that in able-bodied are primarily executed by means of a diverse sense (Bach-y-Rita and W Kercel, 2003). For instance, an electrotactile array laying on the tongue, has been exploited to deliver information coming from two head-mounted accelerometers, in order to stabilize the posture of subjects with bilateral vestibular deficiency (Tyler et al., 2003) or to transfer visual information taken by a camera in blind people (Sampaio et al., 2001).

Direct demonstrations of cross-modal plasticity after training with non-invasive sensory substitution prostheses have been provided. Blind experiencing auditory-to-vision sensory substitution via an ultrasonic echolocation device showed increased occipital cortex activity compared to trained blindfolded sighted control (De Volder et al., 1999), while replacing vision with somesthesis increased the activation of occipital cortex, which correlated with that of posterior parietal cortex (Ptito et al., 2005). Hence, tactile-dependent activation of occipital cortex may occur through feedback projections from multisensory parietal areas.

Heteromodal sensory substitution, relying on cross-modal plasticity, forces the brain towards changes to fulfill the gap between old and new sensory modality. This may involve to fell the replacement as not enough direct and intuitive. An interface able to feedback sensitive information respecting the site and the modality of the cutaneous hand touching and proprioceptive sensations will overcome this issue. We recently demonstrated that the translation of the output of sensors embedded in the prosthesis into patterns of intraneural stimulation allows recognizing shape and stiffness of different objects and consequently choose the appropriate grasp and strength (Raspopovic et al., 2014).

To summarize, cross-modal plasticity has been found to significantly occur in associative areas, such as the parietal cortex, as well as primary sensory areas. Moreover, its occurrence does not only follow compensative mechanisms following brain damage, but may also act as at the basis of sensory substitution.

## Tools use induced plasticity

When we think of human augmentation the image that more probably arises is the one of a man, with additional arm-like devices endowed with tools, who operates in hostile or complex environments. Thus, understanding how brain interacts with tools is mandatory to the present paper.

In the late Seventies Gibson defined the concept of affordance of an object or an environment as “a specific combination of the properties of its substance and its surfaces taken with reference to an animal” in Gibson (1977). “It implies the complementarity of the animal and the environment” (Gibson, 1986). The very external appearance of the tool suggest its unique role in enhancing man-environment relationship: one end of the tool is typically, devoted to the interaction with humans and defines their affordance (i.e., the handle of the hammer) and the other is designed for the interaction with the environment (i.e., the weighted head of the hammer).

Critically, the brain encodes different aspects of the tool, from its more perceptual features to its conceptual meaning and its motor feature. Early knowledge on the cognitive representation of the use of tools in the human brain, and on the putative underlying brain areas, comes from reports of patients affected by brain lesions and suffering from apraxia (Goldenberg, 2003), while only more recently functional imaging studies allowed to infer those processes in healthy subjects (Moll et al., 2000). Moreover, much has been inferred capitalizing on findings from non-human primates. However, humans and monkeys exhibit interspecific differences in the way that their brain deals with tools. In monkey, the mirror neuron system, activated when the animal observes an action performed with the hand, is activated very weakly if the same action is performed through a pincer (Gallese et al., 1996). Visuomotor neurons of premotor area F5 are activated by the visual presentation of a specific tool, or by a subclass of objects, and probably code the motor features of the object (Murata et al., 1997). Visual features of the objects are instead coded by the inferior parietal lobe (Shikata et al., 1996) and in the anterior intraparietal area (Murata et al., 2000). Peculiarly in humans, but not in monkey, the inferior parietal lobule, and in particular the anterior supramarginal gyrus, seems to take part in this network by associating the function of the tool with the required action of the hand (Peeters et al., 2009). Human premotor cortex seems instead to host category-specific representation of tools (Perani et al., 1995), probably as a consequence of the precocious exposition to tools during human motor development.

Either observing, performing or only imagining a task recruits different regions when this is done by means of a tool. A task performed with a tool activates the ipsilateral intraparietal sulcus to a greater extent than the same task performed with the hands (Inoue et al., 2001). Furthermore, the imagination of grasping with tools is accompanied by specific activation of premotor and parietal cortices as well as middle temporal and fusiform gyri (Creem-Regehr and Lee, 2005). In humans, observation of tool use produces a suppression of 20 Hz magnetoencephalographic activity, an hallmark of bilateral primary motor cortex function, which is stronger if the tool is involved in goal-directed actions and if the subject is familiar with the use of that specific tool (Järveläinen et al., 2004).

The neural substrates for the representation of the conceptual knowledge of tools, the ones affected in conceptual or ideational apraxia, are different from the ones hosting the representation of dexterous tool use, affected instead in ideomotor apraxia. Both of them are mainly represented in partially dissociable neural networks, primarily of the left hemisphere (Johnson-Frey, 2004), even in left-handed subjects (Lausberg et al., 1999) and converge in the premotor and parietal areas where the conceptual knowledge of the tool is coupled with the motor program to operate it. Key areas of the tool conceptual knowledge network are the fusiform and the middle temporal gyrus, middle and inferior gyrus, and ventral premotor cortex of the frontal lobe, while dorsal premotor cortex, anterior supramarginal gyrus and intraparietal sulcus of the parietal lobe are activated only if conceptual knowledge is mediated by attention. The network dealing with the motor representation of tools comprises the dorsal premotor and middle frontal gyrus plus the posterior parietal cortex and the intraparietal sulcus (Johnson-Frey, 2004).

Cortical structural changes induced by learning to use a tool can take place rapidly. Learning to retrieve food with a rake in a monkey naive for any tool use produced after only 2 weeks a gray matter increase in the superior temporal sulcus, and in the intraparietal sulcus and bilaterally in white matter underlying the cerebellar cortex (Quallo et al., 2009). On a similar token, gene expression was induced while macaque monkeys learned to use a rake. In particular, during the 2 week period necessary for the acquisition of skillful tool use, but not after the learning phase, an increased level of BDNF and its cellular receptors was found in the anterior bank of intraparietal sulcus, witnessing a learning-induced gene expression, which was linked to the reorganization of visuo-tactile integration in parietal cortex following tool use (Ishibashi et al., 2002).

Finally, as in the case of sensory-motor plasticity reviewed above, the pattern of brain recruitment seems to be specific for a given tool and dependent on previous experience. Imagination of tasks performed with a familiar tool, i.e., a tennis racquet in experienced tennis players, produces a facilitation of corticospinal fibers devoted to the muscles needed to operate the tool, which become more excitable. This process does not take place if the subject imagines motor tasks involving similar tools, either a tennis table-paddle or a golf club, or if the subject is an athlete but not an expert tennis player (Fourkas et al., 2008).

Overall, the above reviewed evidence, speaks in favor of a large representation of tool-use in the cortex. Such representation critically depends upon the conceptual categorization of the tool, its motor mechanics and, importantly, the motor goal that it allows to reach. Such a reach coding of tools and tool-mediated actions is particularly important when it comes to include in the brain representation external augmentation devices. The brain is in facts ready to code for the more conceptual, to the more perceptual and motor features of the newly acquired device for optimal performance.

## Plasticity induced by artifact embodiment

The expansion of the possible interaction between the organism and the environment has been ascribed to the use of tools, while the settlement of the boundary has been ascribed to its practice (Smitsman, 1997). In absence of tools, the part of space where the subject is able to act through his hands and limbs without locomotion, namely the peripersonal space, is coded by dedicated neural structures in the primate brain. A particular relevant role is exerted by visuotactile neurons located in the frontal area 6 and in the inferior parietal lobule (Fogassi et al., 1996). The visual receptive fields of some of those bimodal neurons are arm-centered (Graziano et al., 1994) and surprisingly, following repetitive reaching tasks performed with a rake, increase to cover the expanded range of action of the hand wielding the rake, or increase to encompass the whole length of the tool (Iriki et al., 1996). This seminal finding demonstrated that the human body schema, a mainly unconscious representation of the body arising from the integration of sensory afferences, is not rigid, but maintains a certain plastic flexibility and by integrating sensorimotor inputs (Maravita et al., 2003) can be modulated to embed a tool. Several behavioral data document that how the brain computes tool-use has much more in common with the control of the hands themselves, than that of other objects. Crossing tools affects performance as crossing hands does by producing similar cross-modal interferences and reaching tasks performed with tools are affected in patients with neglect as much as are tasks performed with hands, while, for instance, are spared tasks with pointing devices (Maravita and Iriki, 2004). Moreover a training performed with a grabber extending the range of action, alters the kinematics of subsequent free-hand grasping movements, also reshapes the sensory representation of the arm, inducing subject to localize touch more distally then where it is actually delivered (Cardinali et al., 2009).

**Figure 1:**
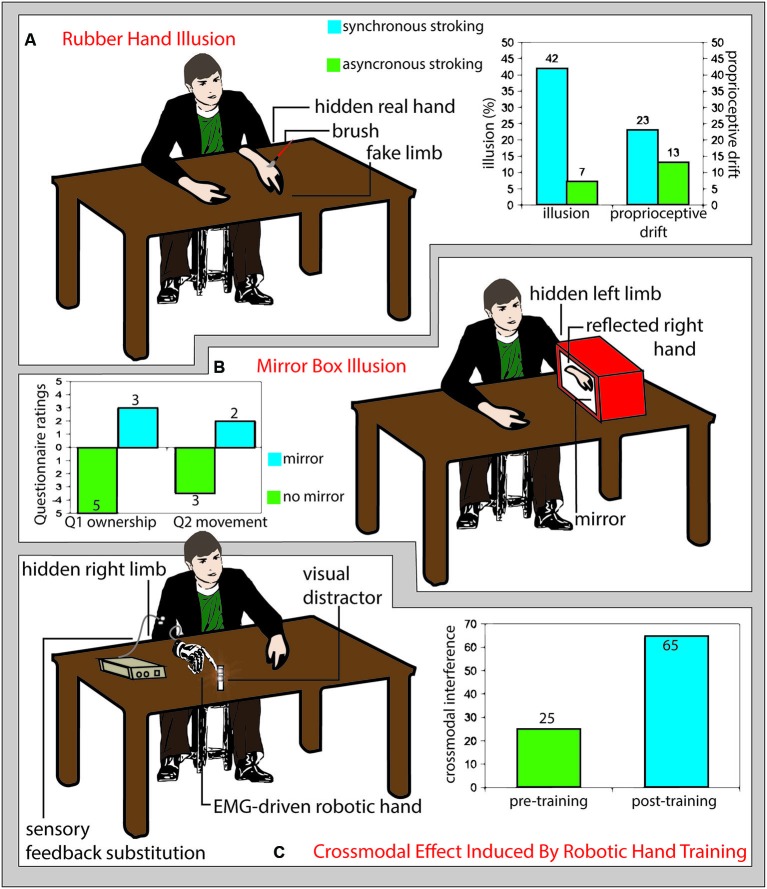
**(A)** Rubber hand illusion. Participants sit in front of a table with their left hand hidden under the table and a fake limb (white detached arm in figure) placed on the table in front of them. If the fake limb is visibly stroked (schematic red brush in figure) together with the real limb (not visible), participants experience the illusion that the touch is referred to the fake limb (illusion) and that their real limb posture shits toward the fake limb (proprioceptive drift). The size of such effects is greater if the touches on the real and fake limb are delivered synchronously (right panel, light blue columns) than asynchronous (right panel, green columns) (Redrawn from the original data of: Botvinick and Cohen, 1998). **(B)** Mirror box illusion. Participant execute right-hand movements while the left arm is hidden from view and kept still inside a box, the right wall of which is replaced by a mirror (Ramachandran et al., 1995). As compared with a no-mirror condition (left panel, green columns), the mirror reflection of the right hand mimics the movements of the left hand inside the mirror box, biasing the participants feeling (assessed through a questionnaire) of ownership (Question 1: “The reflection in the mirror looks like the hand behind the mirror”, left panel, left light blue column) and inducing the illusion of apparent movement (or a true, involuntary, unconscious movement) of the hand inside the box (Question 2: It seems as though the hand behind the mirror is moving; left panel right light blue column) (Romano et al., 2013). **(C)** Crossmodal effects induced by robotic hand training. Prolonged use of an electromyography-driven, detached robot hand (drawn in gray on the right side of the table) providing sensory feedback referred to the participant’s arm (white circles), increased the interference from visual distracter leds located near the robot hand fingers (reddish shadowed circles) tested with the crossmodal congruency paradigm (right panel, light blue columns), as compared to the pre-training assessment (right panel, green columns). This pattern of results suggests a training-dependent expansion of crossmodal integration properties, typical of the peripersonal space near the body, to the space surrounding the robot hand (Marini et al., 2014).

Further than body-space interactions, also the sense of ownership towards external objects may be modulated through experience and external interventions (Figure 1). The rubber hand illusion is a striking example of how our body image can be tricked to embody a replica of a body segment. The vision of a fake hand stroked with a paintbrush synchronously with the stroking of the hidden real hand induces a sense of ownership of the rubber hand and a proprioceptive drift of the perceived position of the real hand towards the fake one (Botvinick and Cohen, 1998; Figure 1A). This process seems to be based on a Bayesian bottom-up integration of convergent multisensory inputs that determines what, within the peripersonal space, belongs to our body (Armel and Ramachandran, 2003) and may involve the activity of multimodal neurons that are activated by proprioceptive, tactile and visual inputs presented in spatial and temporal congruency (Makin et al., 2008). Those neurons have been extensively investigated in non-human primates (Graziano et al., 1994; Fogassi et al., 1996) and their presence has been also documented in humans (Bremmer et al., 2001).

In humans, the rubber hand illusion has been found to activate areas in the premotor cortex (Ehrsson et al., 2004) in the posterior parietal cortex (Ehrsson et al., 2005). Specifically to the mechanisms underlying the illusion, while somatosensory cortex was activated by the rubber-hand situations (comprising asynchronous visuo-tactile stimulation) not inducing embodiment (as assessed by the proprioceptive drift), the occurrence of the RHI was linked to the activation of the posterior insula and frontal operculum (Tsakiris et al., 2007). According to such a different brain substrate, proprioceptive drift and illusory sense of ownership for the rubber hand have been often found to have a low level of correlation (e.g., see Makin et al., 2008; Tsakiris, 2010; Rohde et al., 2011). In particular, the remapping of self body part to the position of an alien hand in external space would include premotor cortex, for the sense of ownership aspect, and the posterior parietal cortex for the monitoring of limb position (Brozzoli et al., 2012). Strikingly, the sense of ownership can be extended even to an empty portion of peripersonal space, thus literally expanding the boundaries of the self, following spatially and temporally congruent visual-proprioceptive signals activating a premotor-intraparietal neural substrate (Guterstam et al., 2013). Finally, brain damage affecting subcortical structures disrupting fronto-parietal connections, may lead to the loss of contralesonal or ipsilesional sensitivity to the RHI (Zeller et al., 2011).

The level of gamma band synchrony over the parietal regions (Kanayama et al., 2009) and a medial shift of the hand representation have also been associated with the strength of the illusion (Schaefer et al., 2009).

When a protocol of repetitive transcranial brain stimulation producing inhibitory effect is delivered over the inferior parietal lobule, it impairs the perceptual component of the illusion, but not the action-oriented component (Kammers et al., 2009), while stroke patients with damage in the white matter connected with prefrontal, premotor and parietal areas have a consistently higher odd to be unable to experience this illusion (Zeller et al., 2011).

However, subjects tested with the rubber hand illusion may experience that the fake hand substitutes their own (Lewis and Lloyd, 2010; Tsakiris et al., 2010) and a sense of disownership toward the real hand (Guterstam et al., 2011). Thus, since the paradigm of augmentation involves the presence of extra-effectors that are controlled in parallel with one’s own limbs, an open question is to what extend the brain may show the ability to host a vivid representation of an extra-limb, while preserving that of the physiological ones.

The mirror box illusion was initially developed to give to amputees a normal, though artificial, visual feedback of their lost limb by reflecting the controlateral healthy one in a mirror. This has been shown to readdress amputees’ aberrant brain plasticity and improve their phantom limb pain (Ramachandran et al., 1995; Ramachandran and Altschuler, 2009). However, also healthy subjects can be induce to feel the sense of ownership for the reflected image by hiding their intact limb inside the mirror box (Figure 1B; Romano et al., 2013).

Reports of humans that, mostly affected by brain lesions located to the right hemisphere, perceive a supernumerary limb in the controlesional side (Halligan and Marshall, 1995) seem to support this possibility. More recently, few modified rubber hand paradigms documented the embodiment of supernumerary limbs, being them two rubber hands for which the subject proved increased protective autonomic response (Ehrsson, 2009) or two virtual copies of the subject real hand (Newport et al., 2010). In the latter case, subjects integrated the perception of both hands into the body image, but were able to control the movement of only one of them, as if only that limb fully integrated in the body representation for action. Incongruence between efferent and proprioceptive signals and between body image and body schema may sustain the possibility to feel a sense of ownership for both the real and the supernumerary limb (Giummarra et al., 2008). Indeed, aberrant plastic modifications of the hand cortical representation that are common in the amputees’ brain (Ramachandran et al., 1992; Flor et al., 1995; Pascual-Leone et al., 1996; Di Pino et al., 2009), not only still allow amputees to experience the rubber hand illusion (Ehrsson et al., 2008), but also to easily embody more than one fake limb at a time (Giummarra et al., 2011). An explanation advanced for this surprising phenomenon is that, in front of multiple, anatomically plausible limbs, the nervous system gradually assigns an equal probability that own limbs may be located at one of the different locations where the fake limbs are, due to multisensory integration (Guterstam et al., 2011).

Which features have to be implemented in the artificial limb to facilitate the process of its embodiment? The level of anthropomorphism of the artifact seems to be a key factor. In front of several objects only realistic prosthetic hands generated strong illusions (Tsakiris et al., 2010), which is prevented by rotating the hand into anatomically implausible postures (Ehrsson et al., 2004). Also proprioceptive afferences play a pivotal role and synchronous active movements of both the real and the fake hand make the embodiment stronger (Tsakiris et al., 2006). Furthermore, the embodiment of virtual hands providing realistic visual input (Slater et al., 2008; Newport et al., 2010).

The embodiment of the supernumerary artificial limb seems like the counterpart of deficit shown by brain-damaged patients who fail to recognize the ownership of their own limbs, attributing them to someone else, and even failing to show anticipatory responses to threatening incoming stimuli (Romano et al., 2014). To the same token, the inclusion of an alien limb in the patient’s body representation could provide several advantages that go well beyond the mere improvement of motor control, including an extend sense of protection against forthcoming threats to the artifact. Indeed, it has been shown that, once a rubber hand is embodied, its threatening induces the activation of the insula and the anterior cingulate cortex that is due to interoception and anxiety, together with a motor activation that reflects the replay of the motor properties of the lost limb. Activity in these regions correlates with the level of embodiment (Ehrsson et al., 2007).

Recently, intracortical recordings in primates have revealed that S1 and M1 are involved in the plastic processes responsible for the embodiment of a virtual hand. The time delay of those responses was compatible with an indirect activation of primary sensorimotor areas by visual cortices, probably trough the fronto-parietal cortical circuitry (Shokur et al., 2013).

In order the rubber hand illusion to arise, congruent tactile and visual afferences are needed. Hence, it is plausible to suppose that to enrich the user experience of prosthesis control with a sensory feedback could be a main determinant to prompt the embodiment of prosthetic limbs. Whole nerve electrical stimulation (Mulvey et al., 2012) and vibrotactile stimulation (brushstroke or stick tapping) (D’Alonzo and Cipriani, 2012) of the real hand are able to substitute a real touch in the processes needed to evoke the rubber hand illusion. A pressure stimulator that translated the data acquired by a load cell mounted on a prosthesis into tactile stimulation of the skin reinnervated with nerves originally devoted to the lost hand was able to evoke the rubber hand illusion for a prosthetic device in patients undergoing target muscle reinnervation (Marasco et al., 2011). Moreover, as it happens for the real hands, the embodiment of the prosthesis should produce a cross-modal integration of tactile afferences with visual stimuli coming from the surroundings of the prosthesis, in order to ameliorate manipulation and sensory anticipation of stimuli in its surrounding environment. In healthy participants, long-term use of an electromyographic signal-driven detached robotic hand, able to provide substitutionary sensory feedback from its fingers via vibrotactile stimulation, produces a pattern of visuo-tactile interference from visual stimuli close to the prosthesis fingers, over tactile stimuli referred to the same fingers, in the cross-modal congruency effect, as typically shown when testing the real hand (Figure 1C; Marini et al., 2014).

However, a lesson learned from the rubber hand illusion is that, further than its intuitive consequences in a more dexterous control of the prosthesis, the more the afferent feedback from a prosthesis is veridical and close to normal physiology, the more the eventual embodiment of the artifact is likely. This has strong implications for the strategy to adopt for prosthesis-user interfacing.

Finally, the integration of neuroprostheses into the dynamic body image of the users, would likely change the body representation in a proficient, but also unnatural way, giving rise to a potential side effect of the prosthesis (Dobkin, 2007). Indeed, the risk of the arising of perceptual distortions of the body image could produce also detrimental effects due to the possible mismatch between the mental body image and the physical body. For example, psychological/psychiatric symptoms may occur, similar to those suffered by teens affected by body dysmorphic disorders, with consequent severe emotional distress, anxiety and depression or to those reported in the body integrity identity disorders or even somatoparaphrenia, where the subject reports extreme discomfort for a body segment that he feel as not belonging to him (Blom et al., 2012; Romano et al., 2014).

## Plasticity induced by the functional replacement of motor output

Amputation is a straightforward model of deprivation-dependent plasticity and the consequences of the use of prostheses may be taken as a model of brain reorganization following the replacement of sensation and motor output. The interruption of incoming and outgoing flow between the lost segment and the brain triggers, in amputees, a plastic rearrangement of pathways and relays, especially in the cortical sensorimotor representation (Ramachandran et al., 1992; Pascual-Leone et al., 1996). The neural underpinning of phantom limb pain has been primarily ascribed to such an aberrant cortical reorganization (Flor et al., 1995). Long-term use of myoelectric (Lotze et al., 1999) or even body-powered (Weiss et al., 1999) prostheses somehow reduces the maladaptive cortical reorganization and the associated phantom limb pain. The right ventral premotor cortex is strongly activated during the control of an EMG-guided prosthesis, while the right posterior parietal cortex activation may underlie its perceptual assimilation in the body schema (Maruishi et al., 2004).

However, interfacing systems relying on the contraction of far spared muscles transmitted through hidden pulleys and cables or superficial electromyographic sensors, may be inadequate for the control of novel multifingered sensorized prostheses and to effectively readdress the aberrant plasticity. To this aim invasive multicontact electrodes have been developed to be implanted in the peripheral nerves (Navarro et al., 2005) and to reopen a bunch of input/output channels directly toward the nervous system of amputees (Micera et al., 2009, 2010). The surgery needed to implant the electrodes, if performed by experts respecting few restrictions, could be considered a low-risk procedure (Di Pino et al., 2013), that may be eventually available in the near future also for healthy people aiming at augmentation.

In parallel with the anthropomorphism and the manipulative skills own by the prosthesis, a key factor that plays a major role in driving the brain reshaping accompanying the employment of a motor substitution device is the achievement of proper solutions for effective and natural bidirectional human–machine interfacing (Di Pino et al., 2009). Indeed, the training for the control of an anthropomorphic dexterous robotic hand interfaced with intraneural multielectrodes with the forearm nerves of the user, induced consistent reversion of the amputation-induced aberrant cortical plasticity. In particular, it can unmask the motor cortical representation of the lost hand (Rossini et al., 2010) and normalize the EEG activation pattern during movement of the phantom hand (Tombini et al., 2012), the functional interhemispheric interaction (Di Pino et al., 2012c) and the cortico-cortical functional connectivity (Ferreri et al., 2014). Those plastic changes are accompanied by a modulation of patient body image, who referred the reshaping of the perceived phantom of the lost limb, now resembling more closely the healthy real arm, and an improvement of his phantom limb pain (Di Pino et al., 2012a,b).

Also targeted muscle reinnervation, the relocation of sensory and motors nerve fibers once devoted to the missing hand toward spared muscles above the line of amputation, could represent a good solution for human–machine interfacing devoted to prosthesis control (Kuiken et al., 2007). Indeed, in amputees this solution results in the return of motor task execution that reversed the previous shift of lost limb cortical motor representation (Chen et al., 2013).

The readdressing of aberrant cortical changes described so far well matches those produced by the transplantation of biological functional body parts. Recovered intracortical and corticospinal excitability was found in patients undergoing toe-to-thumb transfer for their lost thumb (Ni et al., 2010) or for the correct relocation of the functional sensorimotor representation of the grafted hands in bilateral amputees who underwent transplantation of both hands (Giraux et al., 2001). Reacquiring a lost or a new motor output drives brain plasticity also in case of central nervous system damage. Indeed, stroke patients exploiting a mu rhythm-driven magnetoencephalographic brain computer interface (BCI) to operate an orthesis controlling their paretic hand improved their ability to modulate mu rhythm (Buch et al., 2008).

Therefore, the evidence is in favor of a normalization of the aberrant motor cortical plasticity pushed by the reacquisition of a viable motor efference: The more the regained output resembles the previous physiologic condition, the more the normalization of cortical plasticity.

## Augmentation-induced plasticity

Although strictly inherent to human augmentation, the evidence on the evolution of neural plastic processes reviewed so far, is mainly inferred from parallel knowledge acquired from logically related paradigms. From now on, we will focus our discussion on the brain mechanisms that lie behind enhancing able-bodied ability. Augmentation is achieved through BMI, invasive or not, as well as with more traditional devices such as haptic manipulators and vibrotactile stimulators.

Humans have been shown able to acquire new, not-previously experienced, sensory modalities that are instead typical of other animal species. Vibrotactile stimulation can deliver inputs through gyroscopes, accelerometers and magnetometers-embedded in a belt, to be used for space orientation, (Nagel et al., 2005) or for “whisking” through an ad-hoc developed artificial whisker (Saig et al., 2010). Devices for haptic augmented reality are based on the same rationale. Artificial sensors mounted on a fingernail (Ando et al., 2002), on a pen-like tool (Nojima et al., 2002) or directly on an artificial skin layer (Kajimoto et al., 2003) extract from the environment visual information that a tactile display converts mostly into vibrations. A proficient interaction with those devices primarily relies on cross-modal plasticity in the user’s brain. They have been designed to touch the untouchable and can find, for instance, in the enhancement of manipulative skills inside particular surgical theaters their operating field. Surgery under microscope and through robotic effectors can be considered a sort of human augmented scenario, as shown by the ability of transfer to the surgical tool, even a virtual one, multisensory integration properties that are proper of the body itself (Sengül et al., 2012). In humans exposed to an augmented task, the features of the tool adopted for functional augmentation influence the activation pattern of the brain. Indeed, surgeons involved in a laparoscopic procedure manifested higher intrahemispheric sensorimotor EEG coherence, probably because operating straight instruments in a bi-dimensional view requests an enhanced activation of primary and high-order areas. Surgeons performing the same task with the da Vinci® robotic surgical system, which offers more dexterous surgical instruments (EndoWrist) articulated like a wrist in a tridimensional view, had instead higher interhemispheric coherence and a more robust alpha and beta activity, perhaps underlying the enhanced exploitation of bimanuality accomplishing robotic-aided surgery (Bocci et al., 2013).

Proves of the acquisition of unnatural new sensory modalities through invasive brain machine interfaces have been documented in rodents. Rats reorganized their foraging behavior in function of infrared cues sensed by an IR detector directly interfaced with their barrel cortex. Here again, cross-modal plasticity demonstrated to be the key of sensory augmentation. Remarkably, as previously described for the artificial transposition of retinal afferences to S1 that makes S1 responding to light (Métin and Frost, 1989), S1 neurons developed bimodal tactile-IR receptive fields. Unfortunately, it is impossible to disentangle if new inputs were perceived by rats as unnatural stimuli coming from the whiskers or as stimuli arising from a brand new sensory modality (Thomson et al., 2013).

Human augmentation realized some of its best potentialities, also starting to attract a wide attention from non-specialists since brain to machine interfaces have been employed to control artificial limbs, assistive grabbers or wheelchairs. Brain-machine (or computer) interfaces are intrinsic promoters of brain plasticity by forcing an unnatural function of cortical neurons that, instead of modulating the inferior spinal motor neuron, start to be the final nervous element of the motor output chain (Wolpaw, 2007). Moreover, brain machine interfaces give to users the opportunity to have a novel feedback of their brain activity, namely neurofeedback, which would be otherwise unavailable. Such a new form of awareness is a further determinant of brain plastic processes (Dobkin, 2007). The more the feedback on the state of the brain is given in an optimal modality and relayed with good accuracy and delay, the more it is able to support the process of reorganization (Grosse-Wentrup et al., 2011).

The execution of actions through brain machine interface is intentional and goal-directed, since those actions are learned worse if the action-reward contingency is altered or the weight of the reward is reduced (Koralek et al., 2012).

Controlling the output of a BCI, although the controlled task has not prevalent motor features, such as in the case of the control of a visual cursor or the modulation of the pitch of an auditory cursor, seems to be resolved by the brain similarly to a motor task. In this line, the achievement of proficiency is not dissimilar to the one involved in motor skills learning with an initial fast improvement of performance and a later phase of slower learning. In epileptic patients undergoing electrocorticography-monitoring, the control of a one degree-of-freedom BCI by volitional modulation of high gamma band produced a diffuse cortical activation, especially sensorimotor and visuomotor areas. The refinement of performance, achieved through the training, corresponded to a focalization of cortical recruitment, akin to what often seen following motor non-BMI training (Kelly and Garavan, 2005), with a decrement of activity in prefrontal, premotor and parietal cortex, probably due to the shift from a fully cognitive towards a more automatic control of the task (Wander et al., 2013). In a similar paradigm, an increase in non REM spindles has been reported, which witness a facilitation of synaptic plasticity (Johnson et al., 2012). Improvement of BMI control is strongly sustained by an increased striatal to M1 functional coupling (enhanced lower band coherence) and increased firing rate of the cortical-striatal projection. It is based on LTP-dependent plasticity, since mice with defective NMDA striatal receptors exhibit impaired ability to refine their performance (Koralek et al., 2012). The finding of a very similar enhancement of cortico-striatal functional coupling in normal subjects who learn to response with the most appropriate motor behavior to given visual stimuli (Toni et al., 2002) strongly supports the neural correlate correspondence of learning tasks executed exploiting physiological motor outputs or BMI.

A key issue explored in the present paper is whether the brain expand its motor control to a supernumerary limb. In monkeys, several studies documented the ability of the brain to control supernumerary, artificial limbs. Monkey implanted in their primary motor cortex were able to control a 5 degrees of freedom actuated arm for self-feeding, while their own real hand was restrained (Velliste et al., 2008). In a previous study, a significant performance decrease took place when the monkey independently used its own hand (Carmena et al., 2003). To the best of the authors’ knowledge, so far, these findings have not been replicated in humans.

As far as the independency of real and artificial limb control is concerned, cortical motor neurons with augmented outputs, even if still devoted to the control of the natural arm, are able to arrange their activity in order to create what somehow may be considered as the cortical map of the neuroprosthesis. Indeed, in the presence of a constant transformation function of the recorded activity into movement of the external actuator, the learning process results in the formation of a functional neuronal compound, defined by the refinement of tuning parameters such as, preferred directions, mean firing rates and the depth of modulation (Ganguly and Carmena, 2009). The ensemble of neurons controlling the position of a cursor in a 3D space can also plastically adapt its behavior in front of a modification of the transformation function that produces a visuomotor rotation. Both rotated and especially non-rotated units shift their tuning toward the applied perturbation, but rotated units decrease their modulation depth in order to lower their influence on the preferred direction (Jarosiewicz et al., 2008), showing a relative selectivity of response in different subpopulation of neurons. A similar approach, but scaled at the level of entire brain, has been taken by Imamizu and colleagues, that demonstrated an activation of the posterior superior fissure of the cerebellum in subjects relearning to use a computer mouse that underwent a rotational transformation (Imamizu et al., 2000). Learning the use of two mice with alterations of different parameters (rotation and velocity) of their transformation functions activated contiguous, yet different, cerebellar areas (Imamizu et al., 2003). Authors explain cerebellar activity as the result of the formation of a tool-use internal model, a neural process mimicking the input-output flow of tool motor (and probably cognitive) constraints characterizing the interaction.

Amelioration of the intracortical BMI performance also affects the modulation of neuronal firing rate in motor, premotor supplementary motor and parietal regions to a level not directly correlated with the refinement of cursor kinematic. This firing-rate variance showed an inverse *u*-shape trend, increasing in the initial training and decreasing with the acquisition of proficiency, as if it was driven by a progressive reduction of prediction and execution error due, to a progressive refinement of the internal model of the external controlled device (Zacksenhouse et al., 2007). Such a progressive cortical representation of the neuroprosthesis seems to be stable, and ready to use at each new recording session, critical for task accuracy, (since the removal of neurons from the ensemble deeply impairs performance) and resistant to interference since it keeps working even in parallel with the formation of new maps (Ganguly and Carmena, 2009). This body of evidence has enormous implications in favor of the relative stability of the neurons-behavioral links in neuroprosthetic as well as in natural control.

“To resume the section in a few words, controlling external augmenting devices through neural interfaces is resolved by the brain as it does by controlling normal motor output; in particular by building a cortical map of the motor efferences which change its features to achieve, day after day, a more proficient control”.

## Modulating augmentation related plasticity

Along the present manuscript we showed how the propensity of the brain to be plastic can be considered as the fertile soil needed for a proficient implant of new input/output external aid. This implies that any attempt to increase the efficiency of any plastic brain changes or even to redirect them towards the desired direction could result in a more effective blend between the biological and the artificial component of any hybrid bionic system.

In this view, it is known that brain plasticity can be modulated through drugs. Especially noradrenergic agonists have been exploited to enhance M1 excitability (Ziemann et al., 2002), improve motor skill acquisition (Plewnia et al., 2004), learning language (Breitenstein et al., 2004) and in the motor recovery from stroke and other brain lesions (Gladstone and Black, 2000; Schuster et al., 2011). Recently, it has been hypothesized that motor improvement after the administration of amphetamine-like drugs may be due to a better visuomotor integration, with an increased functional coupling between right intraparietal and superior frontal premotor cortex (Grefkes et al., 2010). We already discussed the primary role that the right fronto-parietal circuit plays in augmentation-related plasticity.

Recently, non-invasive neuromodulatory techniques, mostly based on repetitive transcranial magnetic stimulation and transcranial direct current stimulation, have been introduced and showed to effectively inhibit or facilitate the excitability of the motor cortex, possibly through LTP/LTD-like mechanisms (Ziemann et al., 2008). For instance, in humans, transcranial direct current stimulation showed to be able to enhance not only motor skills (Nitsche et al., 2003) and movement speed accuracy (Reis et al., 2009), but also visuomotor coordination (Antal et al., 2004b), learning (Antal et al., 2004a) and frontal functions (Capone et al., 2014). Stimulation of motor cortex has been also successfully used to control chronic neuropatic pain of different etiology, possibly enhancing descending analgesic effects that limit aberrant afferent noxious signals and overall limiting maladaptive plasticity (Andrade et al., 2013; Bolognini et al., 2013a,b). However not invasive neuromodulation can be used also for modulating plasticity of frontal and associative cortices with the aim of cognitive enhancement in the domain of working memory (Andrews et al., 2011; Cantone et al., 2014), problem solution (Chi and Snyder, 2012) and creative intelligence (Cerruti and Schlaug, 2009). Paired associative stimulation is a neuromodulation paradigm that targets specifically sensorimotor integration process by repeating the coupled electrical stimulus to a peripheral nerve and a time-locked TMS pulse to the contralateral M1 (Stefan et al., 2002). Neither paired associative stimulation nor other non-invasive neuromodulatory techniques, perhaps targeting premotor of posterior parietal cortices have been applied, to our knowledge, to attempt to facilitate the embodiment of tools and prostheses. This should definitely deserve our future efforts.

Also less conventional non-invasive brain stimulation can improve augmentation plasticity, as for alpha frequency visual flickering that improves word recall (Williams, 2001). Sleep is a physiological prolonged activity, taking almost a third of our life, when the brain is extremely prone to undergo plastic remodeling especially linked with consolidation of memories (Diekelmann and Born, 2010). A bad sleep is known to negatively impact plastic processes, such as, for instance, those at the base of the recovery from stroke (Zunzunegui et al., 2011). Therefore its modulation could also enhance learning processes related to augmentation. Indeed, it has been shown how sleep-related plasticity can be modulated with transcranial stimulation (Marshall et al., 2006), or simply by delivering external odors (Rasch et al., 2007), with a significant impact on consolidated memory. The tight relation among BCI-related plasticity and sleep is sustained by the local increase of spindles, signs of a cortical state conductive to synaptic plasticity, in subjects trained to control a computer cursor via an electrocorticographic interface (Johnson et al., 2012).

Furthermore, different genetic substrates could have an impact in the individual propensity to be augmented. This can be inferred from the effect of different haplotypes on plasticity related paradigms. Indeed, The Val66Met polymorphism of the brain derived neurotrophic factor, present in about a third of the Caucasian population, has been associated with reduced sensitivity to plasticity-inducing neuromodulation (Cheeran et al., 2008) and with a worse recovery from stroke (Kim et al., 2012). The response of ADHD children to methylphenidate seems to be affected by the Val158Met polymorphism in the Catechol-O-methyltransferase (Kereszturi et al., 2008) and by the genotype of the dopamine transporter (Winsberg and Comings, 1999), which affects also the outcome of working memory training (Brehmer et al., 2009).

Also age-related effect could be taken into consideration as modulating factors for augmentation-related plasticity. Although the rate of enhancement of motor and cognitive ability is maximal at younger ages, when sensorimotor areas express their critical plastic period (Hensch, 2005), there is also evidence that augmentation-related plasticity can take place throughout the entire life span. Plasticity in primary visual (Kaas et al., 1990), auditory (Recanzone et al., 1993) and somatosensory cortices (Merzenich et al., 1984) has been described at later ages and cats deprived of vision during adulthood showed cross-modal improvement of the ability to localize sound, albeit lower than earlier deprived cats do (Rauschecker and Kniepert, 1994).

Finally, can augmentation-related plasticity always be enhanced or it may suffer from ceiling effects that limit the ability to be further augmented? Overtrained athletes can undergo the burnout syndrome (Winsley and Matos, 2011) and excessive use and training can be responsible of aberrant plasticity in sensorimotor areas and in the basal ganglia at the base of the focal dystonia of expert players and musicians (Defazio et al., 2007). There are also reports of ceiling effect in cognitive enhancement (Kwok et al., 2011) and London taxi drivers, whit exceptional navigation ability, acquired worse new spatial memory as if the hyper-representation of posterior hippocampus may undermined new plasticity in the anterior hippocampus (Maguire et al., 2006). However, sometimes improvement can undergo false ceiling effects due to precocious delegation of not yet consolidated functions to brain networks in charge of automaticity (Ericsson, 2007).

## Conclusion

### Summary of the evidence on augmenting-related plasticity

From the body of literature reviewed in the present paper, a few conclusions can be drawn.

First, augmentation-related plasticity takes place at the cellular level, likely through synaptic signals, as evidenced by changes of gray matter thickness and even with neurogenesis in the dentate gyrus.

**Figure 2:**
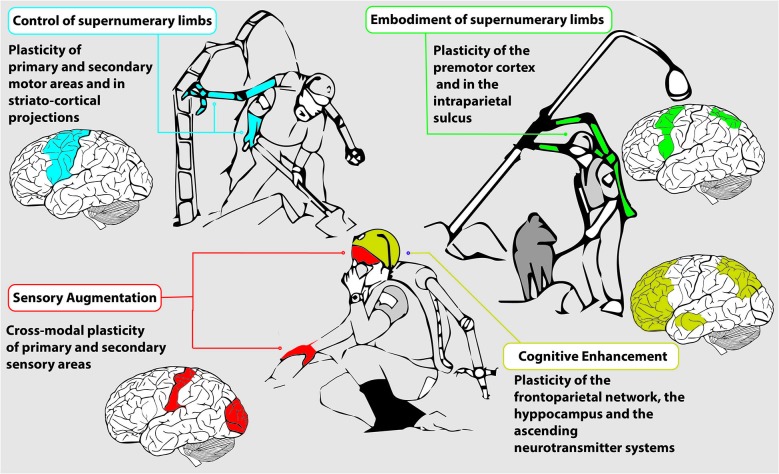
**Possible applicative scenario of human augmentation** Rescuers dug through the rubble of collapsed buildings looking for survivors in the wake of an earthquake exploiting artificial supernumerary limbs (light blue and green), artificial organs of sense (red) and cognitive enhancement (yellow). The most relevant areas of the brain recruited by the task are drown on the side. Control of supernumerary limbs mostly relies on primary and secondary sensorimotor areas and on the facilitation of striato-cortical projection, their embodiment in changes especially taking place in the premotor cortex and in the intraparietal sulcus. Sensory augmentation is enabled by cross-modal plasticity of sensory areas, while cognitive enhancement has in the neural plasticity of the frontoparietal network, of the hippocampus and in the facilitation of the ascending neurotransmitter system its neurobiological substrate.

Second, a number of brain areas have been identified as likely actors of augmentation-based plasticity, playing a role at different stage of the process (Figure 2). The representation of the external world in primary sensory areas is extremely sensitive to activity that modulates their tuning parameters. These cortices are able to accept afferences from different physiological or artificial sensory modalities by shaping the receptive fields of their neurons to make them sensitive to novel kinds of sensory input.

Also M1 is highly susceptible to modulation of cortical representation and corticospinal excitability. The control of neuroprostheses recruits motor and premotor areas, and the acquisition of the skillful use of them promotes the recovery of the cortical representation of a lost limb and its functional interplay with related regions. Training-based skill acquisition gradually decreases attentional recruitment, focusing the activity on sensorimotor areas and increasing the basal ganglia drive of cortical activity. Indeed, the brain can learn to deal with neuroprostheses as it does with normal motor outputs, producing similar learning curves in both conditions.

The frontoparietal network is another functional actor that plays the key role in augmentation-related plasticity. It is strongly recruited in the initial phase of the acquisition of a new motor ability. Premotor cortex is also activated to learn to control an external effector, controls tool motor representation and together with the intraparietal sulcus, which contributes to extracting the visual features of the tool, is the main substrate for artifact embodiment. In these areas, neurons responsible for multisensory integration can be modified to extend their receptive fields and assimilate a supernumerary limb. An anthropomorphic sensorized prosthesis provides the critical sensory afferences needed for a full, comfortable embodiment, and thus optima efficiency, of the artifact. By operating a neuroprosthesis, the brain builds up a cortical representation of the device. This process selectively involves subgroup of interfaced neurons that plastically adapt their firing rate to refine the kinematic parameters and reduce the execution error.

The mirror system of premotor and parietal areas may exert a role in understanding the meaning of an action performed with anthropomorphic augmentation devices and in learning to operate them. Furthermore, plasticity in posterior parietal cortex is responsible also for the assimilation of artificial sensory modalities and for the complex behavioral advantages that from this derive.

Finally, plasticity in the attentional frontoparietal network is the main target of cognitive enhancement, achieved as a corollary effect of sensorimotor augmentation or, selectively, by modulating the neurochemical signals ascending from the brainstem. The hippocampus contributes by undergoing memory-induced changes. The insula and the cerebellum are involved in augmentation-related plasticity too. The cerebellar cortex is activated during the learning of a tool or a neuroprosthesis, contributing to their embodiment; furthermore its activation is related to the formation of an internal model of the external effector. The insula plays a role in the interoception of the embodied artifact and in the relationship of augmented skills with emotions.

### Future perspectives

The evidence provided in this review, unmasks the ability of the central nervous system of primates and humans, not only to master the use of external tools, but also to plastically reshape the body representation and the very sense of the self in favor of a more affordable sensing, and operating in the environment (Clark, 2007).

Central to the topic of the present review is the integration of any augmentative device in the global sense of the self of the user. Operatively, the sense that conscious experience is bound to the self has been defined to emerge from a series of elements including the feeling of body ownership, the perception of self-location in space and the observation of our own body and outside world according to a first-person perspective (Blanke, 2012). The sense of self can be notably disrupted in pathological conditions affecting a single body part such as somatoparaphrenia (the denial of ownership of contralesional limbs, following brain damage) (Vallar and Ronchi, 2009) or the whole body, such as in the out-of-the-body experience phenomena (Blanke and Mohr, 2005). An experimental modulation of the sense of the self for the whole body has been famously demonstrated using the “full body illusion” procedures, in which participants receive tactile strokes, while seeing their own body, filmed by a camera, receiving synchronous or asynchronous strokes of homologous body regions (back/chest). Following this procedure, a variety of illusions of self-identification with the virtual body (Lenggenhager et al., 2007), with the camera viewpoint (as if looking to an alien body), as well as modulation of sensory experience (Aspell et al., 2009; Romano et al., 2014) have been obtained. Different aspects of corporeal self consciousness have been linked to the activation of different brain structures, including premotor, parietal (somatosensory and IPS), extrastriate (EBA) and putaminal regions, as well as the temporoparietal junction, as the result of a process of multisensory integration involving visual, somatosensory and vestibular input (see Blanke, 2012 for review).

The knowledge acquired on the mechanisms of body ownership and, in general, self identification, may put the basis for understanding how plasticity-induced brain augmentation may contribute to the recovery or the enhancement of the sense of the self. The more straightforward situation to think about is certainly the case of amputation. As discussed previously, a key role of functional prostheses is to allow the rebuilt of a full sense of ownership and agency of the prosthesis through a process of training-induced, embodiment (Ehrsson et al., 2008; Marasco et al., 2011; D’Alonzo and Cipriani, 2012; Mulvey et al., 2012). In this respect, the plasticity induced by functional prostheses (Di Pino et al., 2009; Rossini et al., 2010; Maruishi et al., 2004), targets similar sensorimotor brain areas as those modulated by paradigms inducing illusory sense of ownership for alien body parts and could constitute the basis of a full inclusion of external devices into the self as well as the extension of visuo-tactile integration properties to an external augmentation device (Marini et al., 2014).

Indeed, the evaluation of whole brain activity, and the monitoring of cortico-cortical connectivity, for instance by means of functional magnetic resonance, in primates undergoing motor output augmentation through BMI, of which literature to the best of authors knowledge is still wanting, would be of utmost value to depict a comprehensive picture of brain processes underlying augmentation. Such studies could be of out-breaking relevance in order to understand the interplay among different brain structures in the buildup of plasticity, as well as for the monitoring of the neural substrates of possible conditions (pain, emotional distress) that may co-occur as severe side effects.

Finally, we saw how the plastic changes resulting from the interaction with external devices are the necessary neural correlates of functional augmentation, of learning new skills and exploiting artificial senses. Plasticity allows evolving the exploitation of tools through their embodiment and it is strongly correlated with how much the interface constituting the hybrid bionic system is direct and intuitive. We thus propose that, in parallel with more classical instruments for performance monitoring, methods for the functional evaluation of the augmentation-related plasticity, can provide reliable and comprehensive measures of the effectiveness achieved by the hybrid bionic system in accomplishing augmentation.

## Author and Contributors

Giovanni Di Pino chose the topic, conceived the design of the manuscript and wrote the main text. Angelo Maravita was in charge of the sections on tools use and embodiment. Loredana Zollo took care of the section on prostheses. Eugenio Guglielmelli and Vincenzo Di Lazzaro deeply revised the manuscript. All the authors checked and approved the final submitted version of the manuscript.

## Conflict of interest statement

The authors declare that the research was conducted in the absence of any commercial or financial relationships that could be construed as a potential conflict of interest.
